# Storage method of multi-channel lidar data based on tree structure

**DOI:** 10.1038/s41598-022-13138-9

**Published:** 2022-05-31

**Authors:** Hao Chen, Fei Gao, Qingsong Zhu, Qing Yan, Dengxin Hua, Samo Stanič

**Affiliations:** 1 Xi’an University of Technology, Xi’an, 710048 China; 2 Shaanxi Key Laboratory for Network Computing and Security Technology, Xi’an, 710048 China; 3 University of Nova Gorica, 5000 Nova Gorica, Slovenia

**Keywords:** Mechanical engineering, Information technology

## Abstract

The multi-channel lidar has fast acquisition speed, large data volume, high dimension, and vital real-time storage, which makes it challenging to be met using the traditional lidar data storage methods. This paper presents a novel approach to storing the multi-channel lidar data based on the principle of the tree structure, the adjacency linked list, the binary data storage. In the proposed system, a tree structure is constructed by the four-dimensional structure of the multi-channel lidar data, and a data retrieval method of the multi-channel lidar data file is given. The results show that the proposed tree structure approach can save the storage capacity and improve the retrieval speed, which can meet the needs for efficient storage and retrieval of multi-channel lidar data, and improve the data storage utilization and the practicality of multi-channel lidar system.

## Introduction

Lidar, as a new technology of active optical remote sensing detection, has developed rapidly due to its advantages in profile detection with high temporal and spatial resolutions. It has been used for the remote sensing detection of aerosol particle distribution, atmospheric temperature, humidity, wind fields, etc.^1^.

Lidar utilizes use of the atmospheric scattering echo signal(such as Mie scattering of aerosols, Rayleigh scattering, and Raman scattering of atmospheric molecules, etc.), which generated through the interaction of a high-power narrow pulse laser with particles and molecules in the atmosphere, and collected by the telescope to obtain the height distribution of atmospheric parameters, like atmospheric temperature, humidity, wind velocity, aerosol optical properties based on the inversion method of spectral and energy analyses^2,3^. Elastic scattering lidar, hyperspectral lidar, Raman lidar, and differential absorption lidar, as the significant detection technologies and methods, play an extremely significant role in atmospheric remote sensing^4–7^. With the increasing demands of atmospheric remote sensing and environmental monitoring in multi-scale and multi-parameter aspects, lidar develops a comprehensive sensing detection characterized by multiple parameters, long-distance, long time, high precision, and real-time. Therefore, multi-channel lidars that integrate multiple lidar detection technologies are increasingly used for remote sensing detection of atmospheric multi-parameters. It can detect multi atmospheric parameters synchronously by detecting multiple spectral channels. Each channel of multi-channel lidar system has different echo spectrum and experimental information, which increases the difficulty of multi-channel lidar data storage. At the same time, with the increase of the number of channels and the amount of collected data, the demand for fast data storage efficiency and low storage space are also greatly increased.

The storage efficiency of detection data is one of the leading indicators that affect the performance of the multi-channel lidar system. Several storage methods were widely used in recent years, such as character, database, and dedicated format. The character file is the most used for the lidar data storage. It writes the lidar data into a file by text characters or text with delimiter. It usually forms a table or sequential structure with the file formats such as CSV^8,9^, XLS^10^, or TXT^11,12^. This method requires high memory capacity and is only suitable for data access operations with a small amount of data. The database system includes relational databases (such as Oracle, MySQL, SQL Server, etc.) and time-series databases (such as InfluxDB, MongoDB, Cassandra, Couchbase). The time-series database is suitable for large-scale relational and time series data. It is limited in the application of synchronous storage of multi-dimensional data such as spatial–temporal and multi-channel data^13^. Some dedicated format files are designed for the lidar data of a specific detection system with compatibility and scalability limited^14–16^.

So, these storage methods are mainly suitable for the lidar data with a smaller level, simple data structure, and single channel. They have some limitations in the fast storage of multi-channel lidar data, and mainly use characters or floating-point numbers to store data with fixed-length bits and redundant memory space, which requires a lot of memory space and storage capacity. They require frequent data format conversion and storage operations in the process of lidar data storage, and cannot quickly store a large amount of data generated by multi-channel lidar system during operation. In addition, due to factors such as file encoding method, file format definition, and internal relationship structure, the file storage space is large, which leads to the low efficiency in data retrieval and application of multi channel lidar systems.

To solve the above problem of multi-channel lidar data storage, this paper analyzes the multi-dimensional characteristics of multi-channel lidar data, and the data output format of multi-channel lidar system, the hierarchy structure in terms of recording time, channel number, signal intensity, detection distance. Then, we proposal a data storage structure for the multi-channel lidar based on the principle of the tree structure, the adjacency linked list, the binary data storage, and the similar hierarchy between the multi-channel lidar data and tree structure. The practical application result shows that this method can meet the performance requirements of multi-channel lidar data storage in terms of speed and retrieval speed. It improve the data storage utilization and the practicality of multi-channel lidar system.

## Methods

### Characteristics of multi-channel lidar data

At the operation of lidar, a narrow pulse laser beam is emitted from the laser to the atmosphere to interact with the measured parameter target in the atmosphere. Then after the scattered light is received by the telescope with splitting and filtering, the laser echo signal is converted into an electrical signal for subsequent processing. The lidar equation of single scattering is expressed as follows^17^.

1 $$P(r{)} = P0 \cdot Y(r) \cdot \frac{{c \cdot t_{p} }}{2} \cdot \frac{{A_{0} }}{{r^{2} }} \cdot \beta (r) \cdot \exp \left[ { - 2\int_{0}^{r} {\alpha (r^{\prime } ){\text{d}}r} } \right],$$

where *r* is the detection distance (m); *P*(*r*) is the power of echo signal (W), *P*_0_ is initial laser power (W); *Y*(*r*) is a constant between 0 and 1, and it is the geometric overlap coefficient of the optical path between the transmitter and the receiver in lidar system; *c* is the light speed (3 ∙ 10^8^ m/s); *t*_*p*_ is laser pulse width(nm); *A*_0_ is the aperture area of a telescope(cm^2^); *β*(*r*) and *α*(*r*) are the atmospheric backscatter coefficient (km^−1^ sr^−1^) and extinction coefficient(km^-1^) respectively, which are related with atmospheric conditions.

The intensity of lidar data represents the state information of atmospheric parameters at different detection distances along the lidar direction, which refers to the data structure of atmospheric parameter profiles corresponding to the distance point *r*_*i*_ (*i* = 1,2,…,*n*, *n* is the total point number along the detection direction) and the intensity value of laser echo signal *p*_*i*_. Then the data value of the atmospheric parameter at *t*_*j*_ can be expressed as

2 $$v_{j} = \left\{ {\left( {r_{1} ,p_{1} } \right)_{j} {,}\left( {r_{2} ,p_{2} } \right)_{j} {,}...,\left( {r_{n} ,p_{n} } \right)_{j} } \right\},$$

where *j* = 1,2,…,*m*, *j* is the index of lidar data, and *m* is the maximum index number. *v*_*j*_ is called a lidar data unit (LDU), and each LDU is a group of lidar profile data.

In multi-channel lidar system, the specie spectral signals are separated and extracted by a hyperspectral discriminator, it is synchronously recorded in each channel^18,19^. So, the multi-channel lidar data includes the data information such as echo signal intensity, detection range, recording time, channel number, etc.

So, at *t*_*j*_ (*j* = 1,2,…,*m*) within the *k*^th^ channel (*k* = 1,2,…,*q*, *q* is the maximum number of data acquisition channels in the multi-channel lidar system), the laser echo signal data at the distance point *r*_*i*_ can be expressed as

3 $$v_{i,j}^{k} = \left( {r_{i} ,p_{i} } \right)_{j}^{k} ,$$

the multi-channel lidar data *V* can be presented as follows

4 $$V = \left( {\begin{array}{*{20}c} {v^{1} } & {v^{2} } & \cdots & {v^{q} } \\ \end{array} } \right) = \left( {\begin{array}{*{20}c} {v_{1}^{1} } & {v_{1}^{2} } & \cdots & {v_{1}^{q} } \\ {v_{2}^{1} } & {v_{2}^{2} } & \cdots & {v_{2}^{q} } \\ \vdots & \vdots & {} & \vdots \\ {v_{m}^{1} } & {v_{m}^{2} } & \cdots & {v_{m}^{q} } \\ \end{array} } \right),$$

then

5 $$v_{j}^{k} = \left\{ {\left( {r_{1} ,p_{1} } \right)_{j}^{k} ,\left( {r_{1} ,p_{1} } \right)_{j}^{k} , \cdots \left( {r_{n} ,p_{n} } \right)_{j}^{k} } \right\},$$

**Figure 1:**
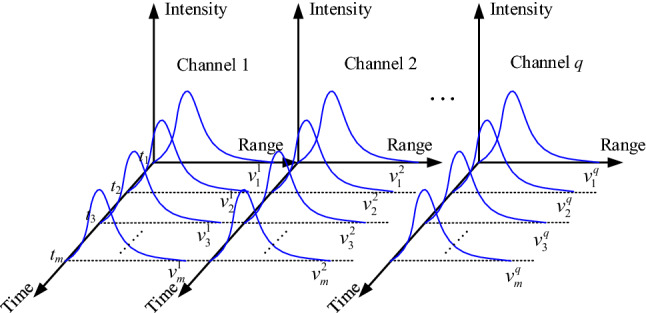
Four-dimensional structure of the multi-channel lidar data.

Each column in data *V* corresponds to the channel unit of the multi-channel lidar data. The four-dimensional structure of the multi-channel lidar data is shown in Fig. 1.

From the above analyses, we know that the multi-channel lidar data consist of several single-channel lidar data. The multi-channel lidar data have adds the channel dimension information to the single-channel lidar data. It can get an LDU in each channel of the multi-channel lidar data, and each LDU has a channel-time relationship. Therefore, the multi-channel lidar data have a large amount of data and a complex structure, the storage methods of single-channel lidar systems do not apply to the multi-channel lidar data.

### Tree structure of lidar data storage

#### Tree structure

The tree structure is a typical nonlinear data structure with a multi-level nested relationship, which is often used to represent the data set with the characteristics of a "one to many" relationship^20^.

**Figure 2:**
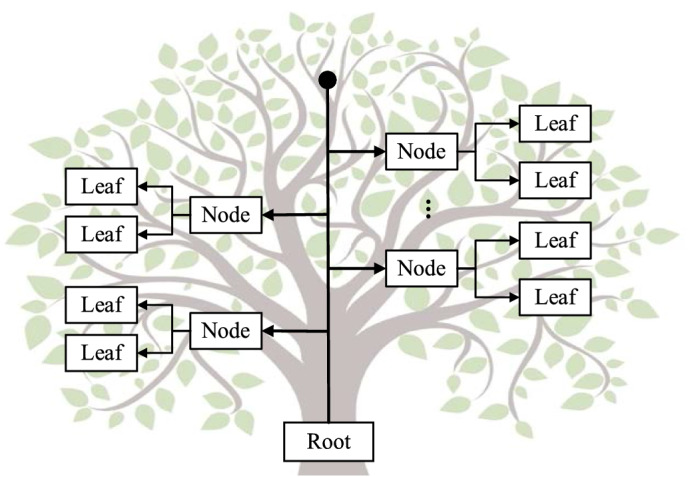
Tree structure.

As shown in Fig. 2, a tree is a limited data set composed of *h* (*h* > 0) nodes. The first node of the tree is a **root**, and node without children is **leaf**. The intermediate node between the **root** and the **leaf** is an internal node. When *h* > 1, the remaining nodes of the tree can be regarded as multiple disjoint finite sets, and each set can be considered as a subtree of the root. The tree structure can classify and sort data effectively, and each node has a unique address. The subtrees are independent of each other, and the operations of the subtree do not affect each other.

The tree structure can clearly express the relationship between data with multi-level and multi-category attributes. It can organize data with the complex relationship efficiently.

#### Tree structure of multi-channel lidar data (TSMLD)

There is a four-dimensional relationship (channel, time, range, intensity) between multi-channel lidar data, and its arbitrary data *vk i,j* shows the different hierarchical distributions in other dimensions.

In general, the multi-channel lidar collects data synchronously from all channels in time. However, the spectral information of laser echo signals and the data properties in different channels are different.

As shown in Fig. 1, at *t*_1_, *t*_2_, …, *t*_*m*_, *q* channels obtain *q* sets of data synchronously, and each collection of data can draw a profile with laser echo intensities. The four-dimensional structure of lidar is similar to the tree structure with the origin of the coordinate system as the root node is shown in Fig. 2, also including the root node **Root**, branch nodes **Node,** and leaf nodes **Leaf**.

**Figure 3:**
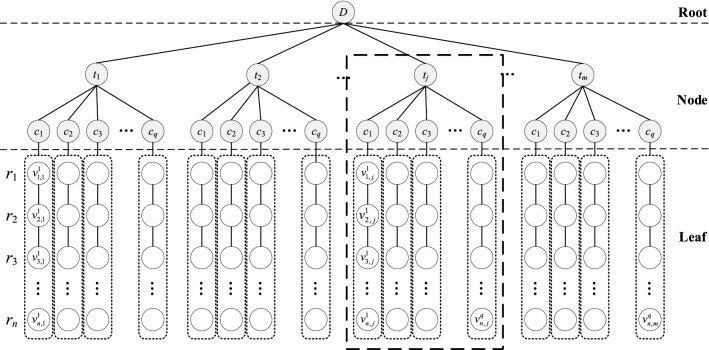
The tree structure of multi-channel lidar data.

To show the hierarchical relationship of multi-channel lidar data, the virtual node sets such as root node, detection time node-set, and channel node-set are introduced, as shown in Fig. 3. The nodes, from first to third layer in Fig. 3 are virtual notes (the gray node). The nodes, from fourth to last layer are the multi-channel lidar data set, which represents the signal data from *r*_1_ to *r*_*n*_ (the white node). Let the first-layer node of multi-channel lidar data be the root node of the tree, denoted as *D*, it corresponding to the **Root** node, and the second-layer nodes are the detection time node sets, indicated as *T*, the third-layer nodes are the channel node sets, indicated as *C*, and the other layer nodes are the echo data node sets, marked as *v*. The second-layer and the third-layer are branch nodes of tree, it corresponding to the **Node** nodes. The detection data nodes from *r*_1_ to *r*_*n*_ have direct internal relationships and consistent data meanings, and they are regarded to form the leaf nodes. It corresponding to the **Leaf** nodes.

The TSMLD shown in Fig. 3 is a connected acyclic undirected graph, denoted as *G*, *G* = {*G*_1_, *G*_2_, *G*_3_,…, *G*_*q*_}, *G*_*j*_ $\in$ *G*, 1 ≤ *j* ≤ *m*, *G*_*j*_ is the subtree of detection time in *G*, representing the data of all channels at the *j*th detection time. The subtree *G*_*j*_ is shown in the dotted box in Fig. 3.

#### The adjacency list of TSMLD

**Figure 4:**
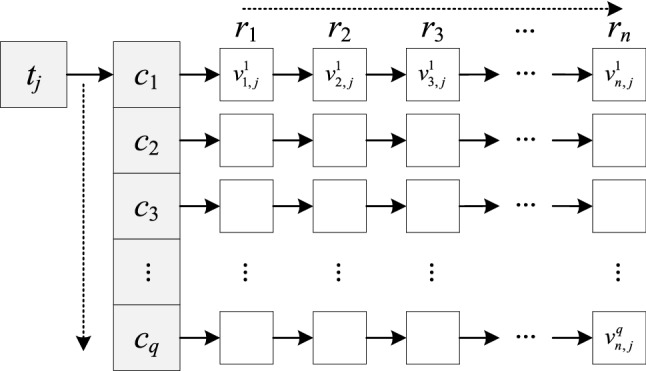
The adjacency list structure of the subtree *G*_*j*_*.*

The adjacency list is the shared storage method for a graph. Based on the hierarchical relationship of the tree structure *G* of the multi-channel lidar data and the structure of the linked table and the array, the paper uses adjacency list structure to represent the storage structure of the multi-channel lidar data. An array requires contiguous memory, it is used to store a small number of nodes, such as the root node, time node, and channel node. A linked list requires distributed storage space, it is used to store many detection data nodes. The node of the tree structure can be retrieved quickly by the subscript of the array and the address of the linked list^21,22^. Therefore, the adjacency list structure of the subtree *G*_*j*_ is constructed as shown in Fig. 4.

The root node of the adjacency list of *G*_*j*_ is expressed as the detection time node *t*_*j*_, and the sub-node of node *t*_*j*_ is the channel node-set. The sub-node of the channel node *c*_*k*_ is the multi-channel lidar echo data set, representing the intensity value of detection data. For the adjacency list of tree structure *G* of multi-channel lidar data, the root node *D* is created, and the subtrees *G*_1_, *G*_2_, *G*_3_, …, *G*_*m*_are added to the sub-nodes of node *D*. The continuous spatial storage is used to deal with the nodes *t*_1_, *t*_2_, *t*_3_,…, *t*_*m*_of *G*_1_, *G*_2_, *G*_3_,…, *G*_*m*_*.*

Therefore, the generation procedure for the adjacency list of the tree structure *G* of the multi-channel lidar data is described as follows. To save memory space, we represent the detection data by binary code.

1. Declare an array of type ***Time**** ajtime*[*m*], let *ajtime*[*m*] = {*t*_1_, *t*_2_, *t*_3_, …, *t*_*m*_};
2. Declare an array of type ***Channel**** ajchannel*[*q*], let *ajchannel*[*q*] = {*c*_1_, *c*_2_, … *c*_*q*_ }.
3. Connect the addresses of the array according to the structure of the subtree *G*_*j*_.

The algorithm is shown in Table 1.

**Table 1:**
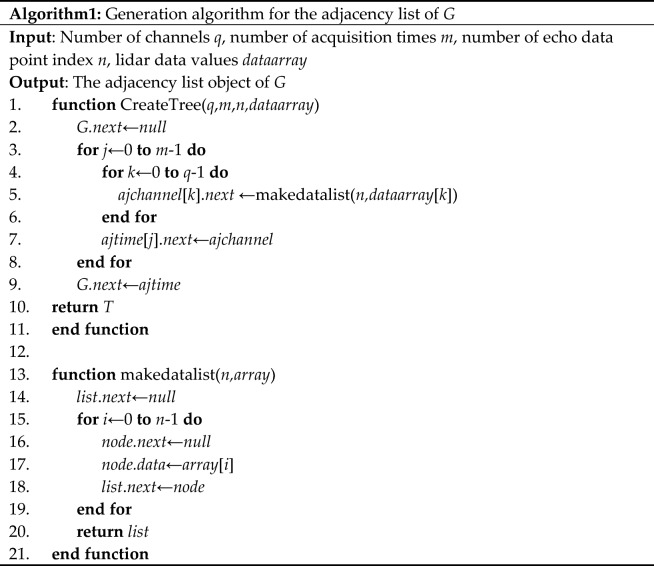
The generation algorithm for the adjacency list of *G.*

The total number of nodes for each multi-channel lidar data is *len* = q*m*n. The time complexity of the generation algorithm for the adjacency list of TSMLD is *O*(*len*), that is, each data node needs to be accessed once.

Next, the tree structure of the multi-channel lidar data is stored by binary code in the adjacency list. Then, it is converted into a data file, and coded by binary using the traversal method of the tree structure.

#### Binary format of TSMLD storage files

Binary formats offer advantages in terms of speed of access. While the basic unit of information is very straightforward in a data file stored in characters (one byte equals one character), finding the actual data values is often harder. This means it is usually necessary to read the entire file to find any value^23–25^.

For binary files, a format description, or a mapping, is required to find the location of any value in the file. However, the advantage of this map is that any value can be found without reading the entire data file.

In addition, in terms of memory, the binary file stored data by numeric format instead of character, and it often requires less memory.

To save the storage space and improve the retrieval efficiency of TSMLD, we present a binary coding file structure of TSMLD. The binary coding file can store many tree structures, so, its structure includes some header information, both for the overall file, and subsections within the file. This header information contains information such as follows:

1. The number of the tree structure *G.*
2. The beginning tag for *G*.
3. The file size.
4. The number of bytes used for each data value (the data value size).
5. The byte location within the file where a set of TSMLD values begins (a pointer).

After the header information, some TSMLDs are stored in the tree segment. Each tree segment is a tree structure *G*, and has a header that includes some information such as the number of detection time nodes, the data size (the length of data segment), and the tree segment number. The back part of the tree segment is the data segment, which contains the data node set of the tree structure *G*.

Each data segment is a subtree *G*_*j*_, and has a header that includes the number of channel nodes *q*, the data size (the length of sub segment), and the data segment number. Behind the header of the data segment are some sub segments.

Each **Leaf** node is stored as a sub segment, and each sub segment consists of data value and header. The data value includes *n* echo data values. Similarly, the sub segment header contains the length of the data value, the record number, etc.

**Figure 5:**
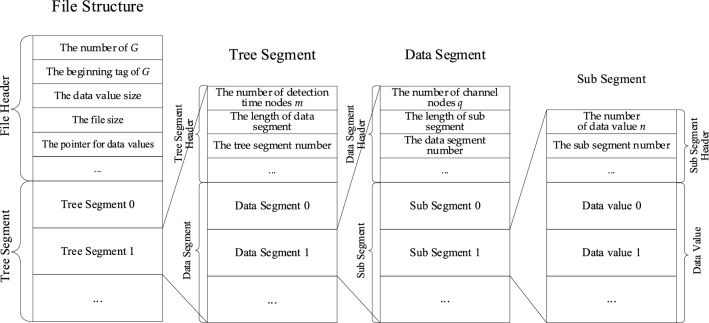
The binary file structure of TSMLD.

The binary file structure of TSMLD is shown in Fig. 5.

#### Storage of TSMLD

In each detection experiment, the data collected from all channels in the multi-channel lidar system constitute a tree structure object of detection time sequence. A tree data storage method (TDSM) *of TSMLD* is given as follows.

**Figure 6:**
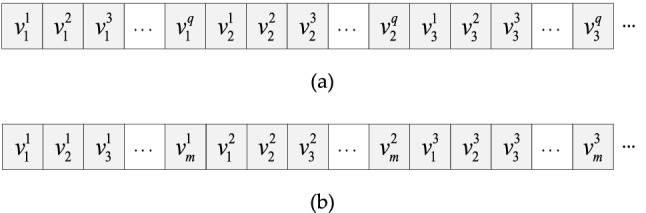
The data sequence of traversal methods. (**a**) The data sequence of TFS; (**b**) the data sequence of CFS.

In a TSMLD, the tree structure *G* must be traversed first. According to the structural characteristics of the tree structure *G*, the traversal methods can be divided into two ways: time-first storage (TFS) and channel-first storage (CFS)^26–28^. The TFS method preferentially stores the data collected by each channel at the same detection time. The CFS method preferentially stores the data collected by each detection time at the same detection channel. The data sequence of the TFS method and the CFS method is shown in Fig. 6.

The detection time information of multi-channel lidar data is given priority in the TFS method. Therefore, the multi-channel lidar data is stored in the order of detection time. The data acquired at a particular detection time is appended to the data received at the previous detection time, and the final data storage sequence is {*v*_1_^1^, *v*_2_^1^,…,*v*_1_^q^, *v*_1_^2^, *v*_2_^2^,…, *v*_2_^q^,*v*_1_^3^, *v*_2_^3^,…, *v*_3_^q^,…}. The detection channel information of multi-channel lidar data is given priority in the CFS method, and the multi-channel lidar data is stored in the order of detection channel. Therefore, the data acquired in each detection channel is appended to the data obtained in the previous detection channel, and the final data storage sequence is {*v*_1_^1^, *v*_2_^1^,.., *v*_m_^1^, *v*_1_^2^, *v*_2_^2^…,*v*_m_^2^, *v*_1_^q^, *v*_2_^q^,…*v*_m_^q^…}.

**Figure 7:**
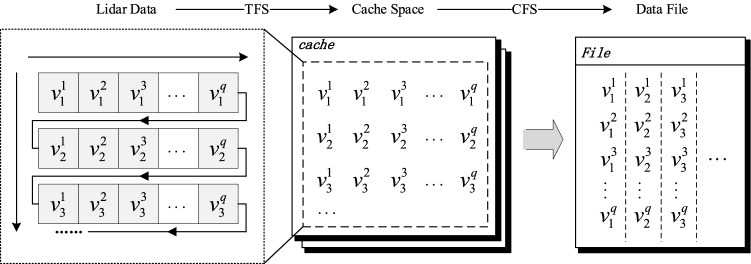
The process of conversion and storage of TSMLD.

The set of multi-channel lidar data $\{ {v_{j}^{1} , v_{j}^{2} , \ldots ,v_{j}^{q} } \}$ (*j* = 1,2,…,*m*) on detection time *t*_*j*_ is consistent with the minimum data storage unit obtained by a single-time multi-channel lidar. In the CFS method, both channel and node tags need to be added to the stored data for data splitting, while only node tags need to be added in the TFS method. So, in data storing and reading, both above methods require additional operation tags to address or split the data, which leads to many redundant operations and reduces storage efficiency. In this paper, a cache storage mechanism is introduced by combining TFS and CFS method, the TSMLD is converted and stored to a data file and the data is coded by binary. The process of conversion and storage is shown in Fig. 7.

The main steps of conversion and storage method are described as follows:

1. Read the binary-coded data $\{ {v_{j}^{1} , v_{j}^{2} , \ldots ,v_{j}^{q} } \}$ at time *t*_*j*_ by time sequence, and write to the cache container *buffer*[*q*].
2. Create cache space *cache*_*a*_, *a* = 1,2…*N*, where *N* is the maximum number of cache space. Write *buffer*[*q*] to *cache*_*a*_ in units of detection time by TFS. Let *l* be the maximum length of *cache*_*a*_ , then, *cache*_*a*_ = $\cup_{j = 0}^{j = l} \left\{ {v_{j}^{1} , v_{j}^{2} , \ldots ,v_{j}^{q} } \right\}$.
3. If the *cache*_*a*_ is full, create cache space *cache*_*a*+1_, and repeat steps (1) and (2) until data collection is completed.
4. Create an array *ldsArray*[*q*][*l*], read *cache*_*a*_ row by row, and store the row data into *ldsArray*[*q*][*l*] by CFS.
5. Write *ldsArray*[*q*][*l*] to the data file *File* coded by binary, and add some header information to the data file, then let *a* = *a* + 1, and go back to step (4).
6. When a > *N*, the TFS and CFS are integrated to store the data cache space.
7. Close the *File* and clear all cache space.

**Figure 8:**
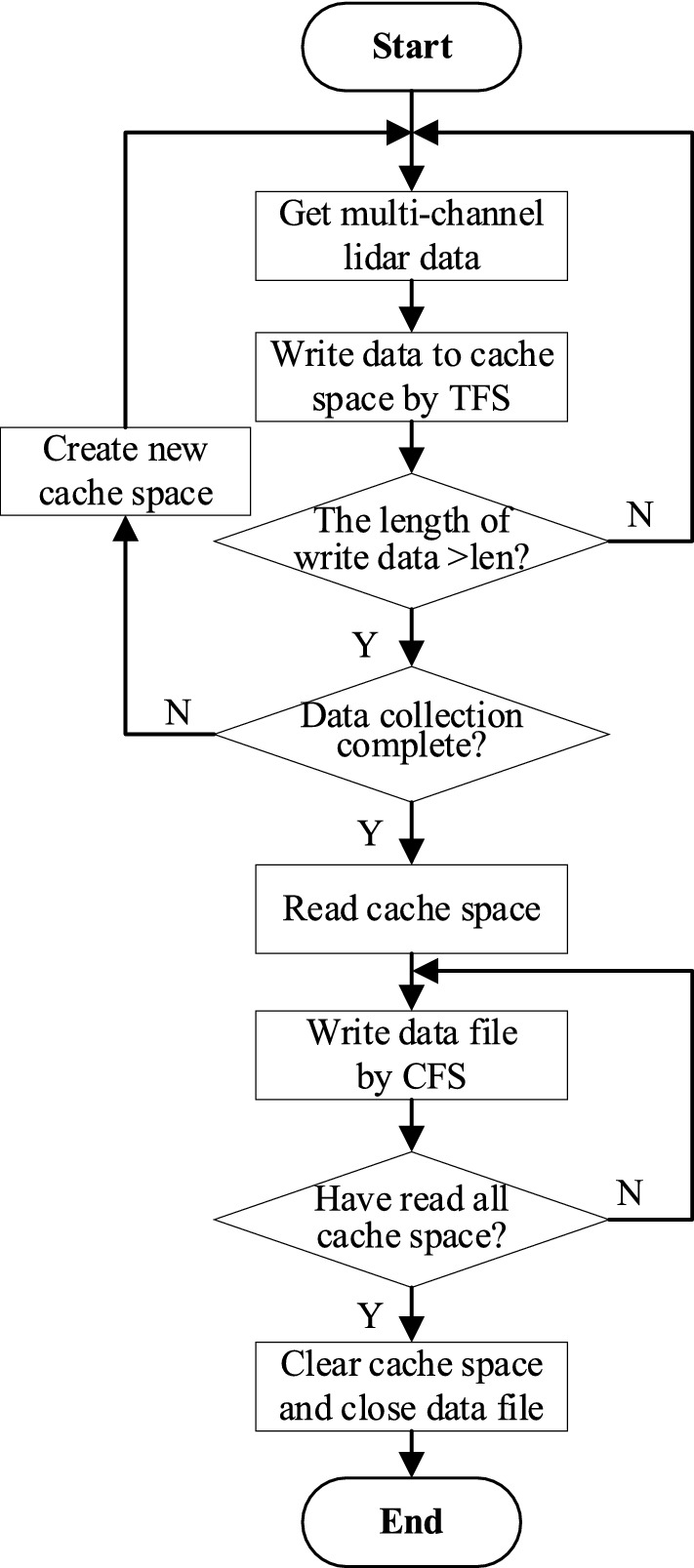
The process of conversion and storage method.

The main process of conversion and storage method is shown in Fig. 8.

By reading the multi-channel lidar data storage files, any data can be retrieved according to the number of channels and time sequence.

Due to the multi-channel lidar data storage files being encoded in binary, so that we can get the details of data by the fix-length byte and the structure of the data file that shown in Fig. 6. Then, any data value can be read by the definition of header information for the data segments, and the data file can be scanned by tree structure of a data file.

The main steps of the data retrieval are described as follows:

1. Load the binary storage file of the multi-channel lidar system, let *bfile* is the file pointer, get the file header length *fhLength* form the file definition.;
2. Read the file header, get the number of tree structure *n*, the file size *sTree*, the beginning pointer *bPointer*, etc.
3. Read tree segment by index; let *i* = 0;
4. Read the *i*-th tree segment, that is the *i*-th tree, donated as *T*_*i*_;
5. Read the header information of *T*_*i*_., get the number of detection time nodes *m*, the length of data segment in *i*-th tree segment,etc.
6. Read data segment by index;
7. Let *j* = 0, get the *j*-th data segment, that is the sub tree *G*_*j*_ of *T*_*i*_ .
8. Read the header information of *G*_*j*_, get the number of detection channel nodes *q*, the length of sub segment in *j*-th data segment, etc.
9. Read sub segment by index, let *k* = 0,
10. Get the *k*-th sub segment, that is the data list in detection channel *c*_*k*_ of *G*_*j*_ .
11. Traverse the data value of channel *c*_*k*_ according to the storage method of the adjacency list;
12. If *k* < *q*, let *k* = *k* + 1, go back to (10);
13. If *k* = *q*, *j* < *m*, let *j* = *j* + 1, go back to (8);
14. If *j* = *m*, *i* < *n*, let *i* = *i* + 1, go back to (5);
15. Close the *bfile*.

**Figure 9:**
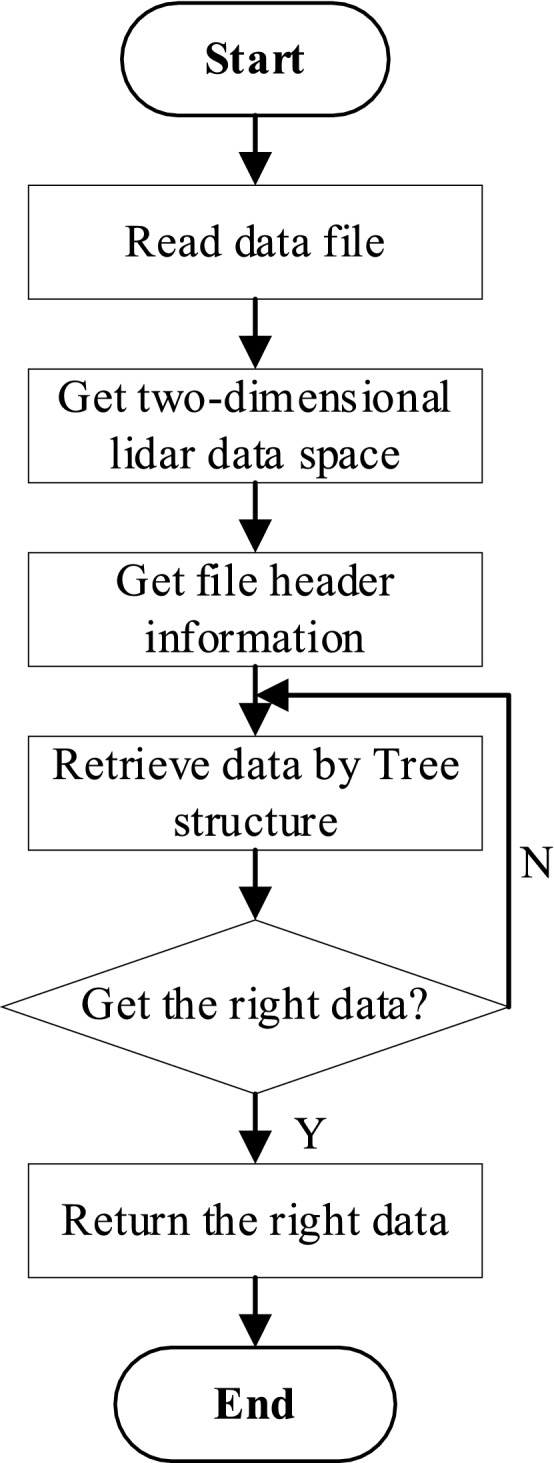
The process of data retrieval.

The data retrieval process is shown in Fig. 9.

## Experimental

**Figure 10:**
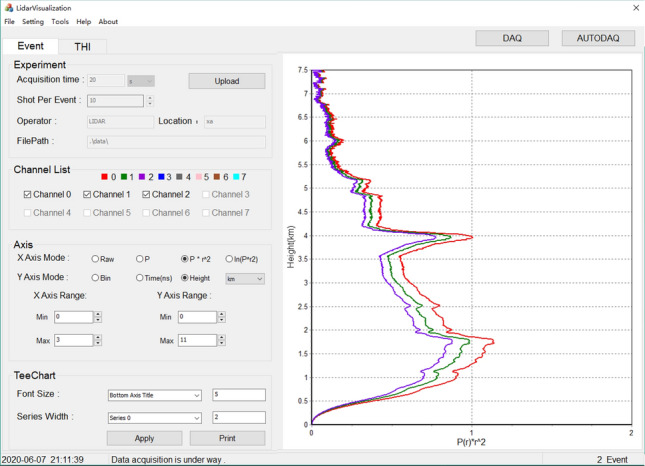
The interface of the software system.

The experimental data comes from the ultraviolet Raman lidar system in the Center for Lidar Remote Sensing Research of Xi'an University of Technology^29^. In the experiment, an industrial control cabinet Pxie-1071 and a data acquisition card Pxi-5105, developed by NI company, are used as data acquisition equipment. their main parameters are shown in Table 2. The storage and retrieval experiment for lidar echo data is performed under the multi-channel mode. The hardware system and the user interface of the software system for data acquisition are shown in Fig. 10.

**Table 2:** The main parameters in the experimental platform.

Parameters of data acquisition card	Value
Channel number of single acquisition card	8
Maximum real-time sampling rate	60 MS/s
Signal input range	0–30 V
Built-in memory of board card	512 MB
Maximum single acquisition length (8-channel mode)	16,384
CPU model	i5-4200
Dominant frequency ofCPU	2.3 GHZ
Disk space	48G SSD
Built-in memory	8 GB
Maximum system bandwidth	3 GB/s

## Results

In the storage capacity test, we consider four storage methods in Sect. 1. There are two method in character file, the text sequence storage method (TSM)^30,31^ and the table structure storage method (TSSM)^32,33^. The data file contains only character or text in the TSM, and text with delimiters in the TSSM. The text with delimiter can be divided into table structure by delimiter. The database storage method (DSM)^34^, and the tree data storage method (TDSM) given in this paper also to be considered. A detailed comparison of these four storage methods is conducted regarding the storage capacity and retrieval speed of the multi-channel lidar data. The data in the TSSM, TSM, DSM, and TDSM are stored in table format files, text files, MySQL database and binary files, respectively.

**Figure 11:**
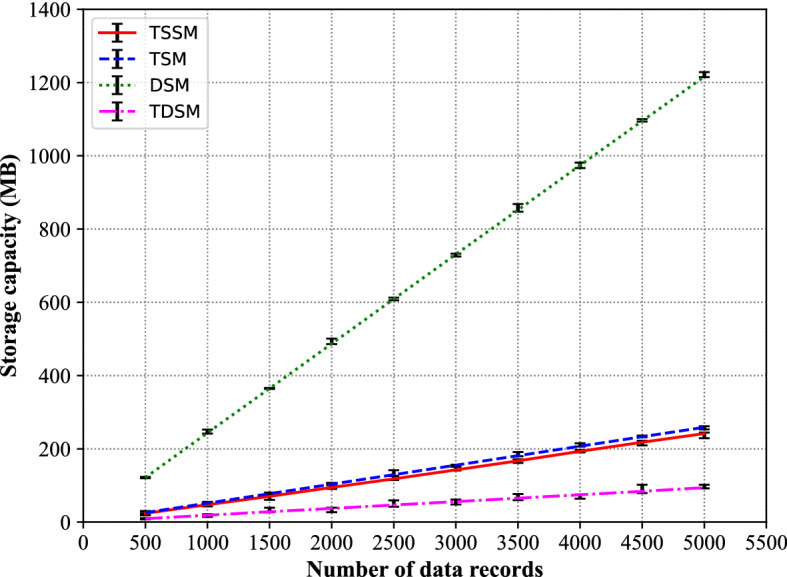
Comparison of storage capacities between TSSM, TSM, DSM and TDSM.

Figure 11 presents the variation of the file storage capacity of the multi-channel lidar echo data with the four storage methods. Table 3 presents the test data in the file storage capacity of the multi-channel lidar echo data with the four storage methods.

**Table 3:** Detailed information of storage capacities between TSSM, TSM, DSM and TDSM.

Number of data records	TSSM	TSM	DSM	TDSM
500	24.12	25.91	121.65	9.43
1000	47.53	51.82	243.74	18.72
1500	70.34	77.52	364.85	28.42
2000	94.66	103.61	486.95	37.31
2500	118.15	129.53	609.01	46.73
3000	142.57	155.44	731.01	55.92
3500	167.38	181.31	852.01	65.43
4000	193.24	207.23	974.01	74.6
4500	218.28	233.12	1095.01	84.11
5000	241.71	258.82	1216.01	94.12

As Fig. 11 and Table 3 shows, with the increase of multi-channel lidar data, the storage capacities of TSM and TSSM are almost the same and increases linearly, since both are character-based storage, and the storage capacity of each character is fixed. The DSM has the largest storage capacity because the structured approach is utilized to improve the retrieval speed in the MySQL database system Still the building of data indexes in the relational model results in the multiplied increase of storage space. With the same data volume, the TDSM has the minimum storage capacity owing to the compressibility of the binary storage method in comparison to the text character method. The text character method focuses on the distribution of storage space for each character, while the binary data aims to compress and store all the data into a more compact file with more space saved in the meanwhile.

**Figure 12:**
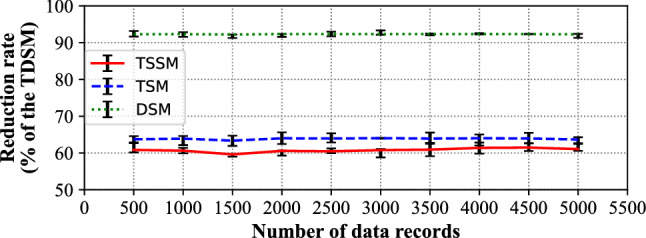
The reduction rate of storage capacity of TSSM, TSM and DSM.

Figure 12 and Table 4 shows the storage capacity reduction rate of the TDSM compared with the TSSM, TSM and DSM. The TSSM and TSM have similar trends in the reduction rate of storage capacity, ranging from 60 to 64%. However, the DSM with the maximum storage capacity has a significant reduction rate of about 92%.

**Table 4:** Detailed information of reduction rates of storage capacity to TSSM, TSM and DSM.

Number of data records	TSSM	TSM	DSM
500	60.83	63.71	92.27
1000	60.63	63.90	92.33
1500	59.60	63.35	92.22
2000	60.57	64.00	92.34
2500	60.46	63.94	92.33
3000	60.77	64.03	92.35
3500	60.91	63.93	92.32
4000	61.39	64.00	92.34
4500	61.47	63.96	92.33
5000	61.07	63.64	92.26

**Figure 13:**
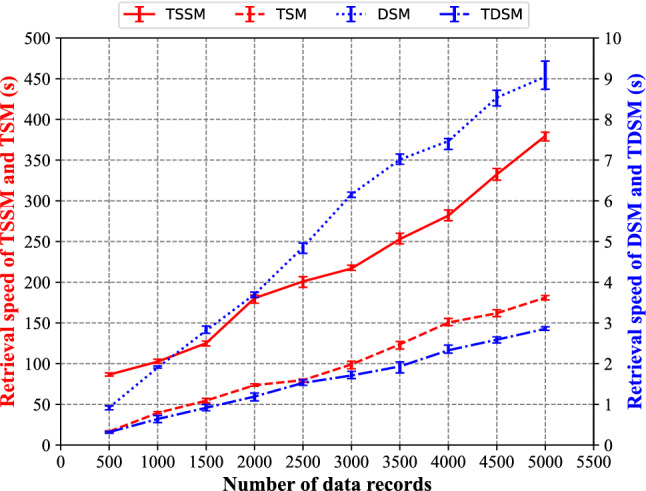
Comparison of data retrieval speed between TSSM, TSM, DSM and TDSM.

In the retrieval speed test, we mainly test the multi-channel lidar data retrieval speed of four methods for 1000 random retrieval visits under different data volumes. The test result is shown in Fig. 13 and Table 5.

**Table 5:** Detailed information of data retrieval speed between TSSM, TSM, DSM and TDSM.

Number of data records	TSSM	TSM	DSM	TDSM
500	86.53	16.65	0.92	0.32
1000	102.46	39.44	1.90	0.64
1500	125.14	54.56	2.81	0.92
2000	180.45	73.60	3.70	1.19
2500	200.95	79.74	4.86	1.53
3000	216.84	99.07	6.16	1.71
3500	252.99	123.76	7.01	1.93
4000	281.83	150.52	7.47	2.33
4500	332.45	161.91	8.54	2.59
5000	379.69	181.13	9.05	2.86

From Fig. 13 and Table 5, we can find that the TSSM is the most time-consuming method, followed by the TSM, and the time consumption of DSM and TSDM methods is kept at a low level with the least time of fewer than 10 s. With the increase of multi-channel lidar data, there is a linear increase in the data retrieval time of TSSM and TSM methods since a linear increase is also shown in the data storage capacity, and the data retrieval is linearly correlated with the data volume. Similarly, the TSSM, with the rise of multi-channel lidar data, is affected by the reading and writing speed of I/O and the retrieval speed of characters. It leads to an apparent reduction in retrieval speed and an increase in time consumption. Based on the professional database management system, the DSM uses the structured approach to deal with field data and create indexes for field data. Despite the increase in data storage space, a noticeable optimization effect is shown in the improvement of data retrieval efficiency. The time of data retrieval of the DSM is less than 10 s, and the TDSM takes less than 5 s in the experimental test. Due to a combination of the tree structure traversal method and binary coding, the data at any position in the data file can be quickly read based on the detection time and the channels. The process is less affected by the amount of data, and it saves the time of data retrieval. In other words, this method reduces the time consumption of multi-channel lidar data storage. In addition, the large amount of multi-channel lidar data needs less memory to be the buffer during storage.

**Figure 14:**
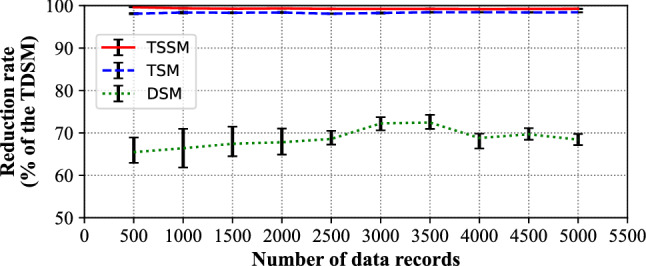
The reduction rate of data retrieval time of TSSM, TSM, DSM.

By comparing with the TSSM, TSM and DSM, Fig. 14 and Table 6 shows the reduction rate of data retrieval time based on TDSM. It turns out that the reduction rate of TDSM reaches 98% because of the apparent improvement of retrieval efficiency compared to the TSSM and TSM.

**Table 6:** Detailed information of reduction rates of data retrieval time to TSSM, TSM, DSM.

Number of data records	TSSM	TSM	DSM
500	99.63	98.09	65.47
1000	99.38	98.38	66.38
1500	99.27	98.32	67.45
2000	99.34	98.38	67.82
2500	99.24	98.08	68.58
3000	99.21	98.27	72.24
3500	99.24	98.44	72.45
4000	99.17	98.45	68.81
4500	99.22	98.40	69.68
5000	99.25	98.42	68.43

In addition, the data retrieval time of the TDSM and DSM remained at a low level, with a decrease of about 70%, which fluctuated between 65 and 72% compared to the DSM.

There is a multi-channel lidar data set, the length of the multi-channel lidar data is *len* = q*m*n, *q* is the number of the channel, *m* is the number of the detection time, *n* is the number of the data value. In the TDSM, TSSM, TSM, each data is accessed at least for once, their time complexity is *O*(*len*). In the DSM, the multi-channel lidar data is stored in three tables at least, channel table, time table, and data table, the number of rows is *q*, *m*, *n*. Normally, each data should be traversed, and the time complexity is *O*(*len*). However, if the data index is not created in database, the time complexity is *O*(q*log(*q*) + m*log(*m*) + n*log(*n*)).

## Discussion

The software system for data acquisition of multi-channel lidar system integrates the TSSM, and the programming language is C++. Due to technical limitations, it can only run-on the windows series operating system. The operating system used in the experiment is Windows 10. However, a complete multi-channel lidar system contains multiple functional subsystems. The control subsystem controls the hardware devices in the multi-channel lidar system, and it usually runs on Linux systems such as Ubuntu and Debian. If the data acquisition system and the control software system can be integrated and run across platforms, the work efficiency of the multi-channel lidar system can be further improved. The cross-platform operation of the data acquisition system requires drivers for different operating systems to connect to the data acquisition card and the other programming language or software framework is used to program the data acquisition system. But the replacement of operating systems and programming languages will inevitably affect the performance of the data acquisition system. How to be involved or to be affected by what factors, that will study in the next research work.

## Conclusion

Through the analysis of relational characteristics and storage requirements for lidar data, the present paper develops a storage method for the multi-channel lidar data based on the tree structure for the multi-channel lidar system. Drawing on the hierarchical relationship structure of the channel, time, and range of multi-channel lidar data, this method combines the linked list and the adjacency list with an array structure to construct the storage method, and the multi-channel lidar data is encoded by binary code in the adjacency list. Finally, the multi-channel lidar data is stored in binary format files. This study can be used to build data processing and storage systems for the multi-channel lidar system or similar systems. In addition, it can be an example of a solution to a similar lidar system when a selection from a list of alternatives is required. The experimental results show that this method, compared with the traditional list structure and the text character storage method, can save at least 60% of the storage capacity and increase the retrieval speed by about 98%. The superior advantages of the technique lay a solid foundation for the effective use of multi-channel lidar data.

## Data Availability

All materials and data used should be available at Xi’an University of Technology/China. The data used to support the findings of this study are available from the corresponding author upon request.
